# Evaluation of Atrial and Ventricular Myocardial Repolarization Markers During Acute Migraine Attack

**DOI:** 10.3390/jcm15103952

**Published:** 2026-05-20

**Authors:** Yavuz Katırcı, Emine Emektar, Meral Yıldırım, Özge Güler, Osman Korucu, Yücel Yüzbaşıoğlu, Mesher Ensarioğlu, Süleyman Mehmetcan Ceritoğlu, Onur Küçük, Semih Aydemir

**Affiliations:** 1 Department of Emergency Medicine, Gülhane Training and Research Hospital, 06010 Ankara, Turkey; 2 Department of Emergency Medicine, Atatürk Sanatoryum Training and Research Hospital, 06290 Ankara, Turkey; 3 Department of Emergency Medicine, Medicalpark Gebze Hospital, 41400 Kocaeli, Turkey; 4 Department of Neurology, Atatürk Sanatoryum Training and Research Hospital, 06290 Ankara, Turkey; 5 Department of Anesthesiology and Reanimation, Gülhane Training and Research Hospital, Health Sciences University, 06010 Ankara, Turkey; 6 Department of Anesthesiology and Reanimation, School of Medicine, Trakya University, 22030 Edirne, Turkey; 7 Department of Anesthesiology and Reanimation, School of Medicine, Dokuz Eylül University, 35330 İzmir, Turkey; 8 Department of Anesthesiology and Reanimation, Yenimahalle Training and Research Hospital, Yıldırım Beyazıt University, 06370 Ankara, Turkey; drsemihaydemir@gmail.com

**Keywords:** migraine, autonomic nervous system, electrocardiography, arrhythmias, cardiac, P-wave

## Abstract

**Background**: Migraine is a neurological disorder affecting approximately 15% of the general population, and autonomic nervous system (ANS) dysfunction is a well-characterized feature of the condition. Sympathovagal imbalance during acute migraine attacks has been linked to cardiac electrical instability. This study aimed to evaluate atrial and ventricular myocardial repolarization markers in migraine patients at three serial electrocardiography (ECG) time points. **Methods**: A prospective observational cross-sectional study was conducted in a tertiary emergency department (ED), enrolling 70 migraine patients and 70 age- and sex-matched healthy controls. Three 12-lead ECGs were obtained per patient: during the migraine attack (within 60 min of ED admission), after analgesic treatment (verbal pain relief or Numeric Rating Scale [NRS] decrease greater than 4 points), and in the pain-free period (at least 24 h after the attack, within 7 days). Measured parameters included P-wave duration, P-wave dispersion, QT interval, QT dispersion, corrected QT (QTc) duration (Bazett formula), QTc dispersion, Tpeak–Tend (Tp-e) interval, Tp-e dispersion, and Tp-e/QTc ratio. ECGs were evaluated by two blinded emergency medicine specialists. **Results**: All repolarization markers were significantly higher in migraine patients than in controls (*p* < 0.05 for all). Comparing the first (ictal) with the second (post-treatment) measurements, all markers except P-wave dispersion decreased significantly (*p* < 0.05). All markers were significantly higher during the attack than in the pain-free period (*p* < 0.05 for all). Tp-e interval and Tp-e/QTc ratio showed a further significant reduction between the second and third measurements (*p* = 0.016 and *p* = 0.004, respectively). P-wave dispersion was significant only for the first-to-second comparison (*p* = 0.034) and did not differ significantly between the first and third (*p* = 0.137) or second and third (*p* = 0.725) measurements. Pulse rate was significantly higher in the migraine group than in controls (*p* = 0.012). **Conclusions**: Acute migraine attacks were associated with significant elevation of both atrial and ventricular repolarization markers, with near-normalization during pain-free periods. These findings are consistent with a proposed mechanism of ANS-mediated cardiac electrical instability during acute attacks, although direct confirmation in future studies is required. Clinicians managing acute migraine in the ED should consider ECG monitoring in patients with cardiovascular risk factors. For anesthesiologists and intensivists, the elevated Tp-e and Tp-e/QTc observed ictally indicate that preoperative ECG assessment in migraine patients may be warranted to guide anesthetic planning.

## 1. Introduction

Migraine is a common neurological disorder, affecting approximately 15% of the global population and exhibiting a marked female preponderance [1]. Its cardinal features extend beyond headache. Nausea, cutaneous allodynia, photophobia, and phonophobia arise from central sensitization of trigeminovascular and brainstem pathways rather than from direct autonomic activation. Alongside these core features, migraine attacks are frequently accompanied by cranial autonomic symptoms such as lacrimation, conjunctival injection, nasal congestion, and aural fullness, which occur in roughly half of pediatric and adult migraineurs and reflect parasympathetic trigeminal–autonomic reflex activation [2]. Systemic autonomic involvement is also a recognized feature of the migraine phenotype, with associated changes in cardiovascular regulation, sudomotor function, and gastrointestinal motility [3]. A systematic review and meta-analysis of 692 participants reported an association between migraine and impaired sympathetic and cardiovagal function across all four standardized ANS tests, with the largest effect observed for sympathetic function during isometric challenge; the authors hypothesized that peri-ictal autonomic differences may be more pronounced than the interictal deficits captured in controlled testing conditions [4]. Whether this association reflects a shared predisposition, a consequence of recurrent attacks, or an active mechanism during the ictal phase remains incompletely characterized, and its cardiovascular implications are not yet defined.

Electrocardiographic (ECG) abnormalities have been documented during acute migraine attacks across multiple cohorts. The only published meta-analysis of ECG parameters in migraine, pooling 667 patients and 208 controls across 13 studies, reported that QTc interval was significantly longer during attacks than in pain-free periods, with a 6.23-fold higher risk of QTc prolongation during attacks [5]. The 13 included studies did not consistently report or control for triptan or other analgesic administration around the time of measurement, which limits attribution of the observed changes to migraine pathophysiology alone. Aygun et al. reported that 30% of 30 migraine patients showed rhythm abnormalities during attacks, with mean ictal QTc reaching 442 ms and 40% exceeding the 440 ms threshold during attacks versus none in the pain-free period [6]; the analgesic regimen in that study was described as non-narcotic but not further specified. Ventricular tachycardia during a basilar-type migraine attack has also been reported in a patient with angiographically normal coronary arteries and structurally intact myocardium [7]. Cross-sectional data describe a pooled atrial fibrillation (AF) prevalence of 1.39% in migraine patients, rising to 1.61% in those with aura [8]; the authors of that analysis note that this absolute prevalence is low, consistent with the young age distribution of the included migraine cohorts. Longitudinal data tell a different story. Adelborg et al. followed 51,032 migraine patients matched 1:10 with the general Danish population for up to 19 years and reported an adjusted hazard ratio for AF or atrial flutter of 1.25 (95% CI 1.16–1.36), with a stronger association in migraine with aura (HR 1.31, 95% CI 1.11–1.55) than without aura (HR 1.16, 95% CI 0.99–1.35) [9]. Together, the low cross-sectional prevalence and the elevated long-term risk are compatible with an electrically vulnerable atrial substrate that has not yet manifested as overt arrhythmia in this comparatively young population. Taken across these reports, migraine attacks are associated with measurable changes in cardiac electrical activity; the extent to which these changes reflect migraine pathophysiology rather than acute pharmacological effects of analgesics, including triptans, has not been systematically addressed in prior studies.

Repolarization dispersion markers provide a more granular assessment of arrhythmic risk than QTc alone. The Tpeak–Tend (Tp-e) interval reflects global ventricular repolarization dispersion, incorporating both transmural and apicobasal gradients, with in vivo validation confirming that Tpeak coincides with the earliest and Tend with the latest ventricular end of repolarization [10]. Prospective cohort studies have independently associated rate-corrected Tp-e with ventricular tachyarrhythmia and all-cause mortality, demonstrating superior predictive value over QTc alone [11], and ROC-validated thresholds for Tp-e and Tp-e dispersion have been established as high-risk markers for life-threatening ventricular arrhythmias [12]. Sympathetic hyperactivation increases transmural dispersion of repolarization (TDR) and predisposes to arrhythmias, an effect further amplified by pain-induced parasympathetic suppression [13]. Atrial repolarization heterogeneity carries analogous clinical relevance: P-wave dispersion, defined as the difference between maximum and minimum P-wave duration across all 12 leads, is an established predictor of AF in coronary artery disease, hypertension, and sleep apnea [14,15], and has been shown to be significantly elevated in migraine patients in the absence of overt cardiovascular disease [16].

Despite this accumulating evidence, prospective data combining Tp-e, Tp-e/QTc ratio, Tp-e dispersion, and P-wave dispersion across serial ECG time points in an emergency department (ED)-based migraine cohort are limited. The only published ED-based study measuring Tp-e during acute migraine reported a significantly prolonged Tp-e interval with an elevated Tp-e/QTc ratio, whereas QTc did not differ significantly between groups, underscoring Tp-e as a more sensitive marker than QTc in this setting [17]. None of the 13 studies included in the only published ECG meta-analysis reported Tp-e interval, Tp-e dispersion, or Tp-e/QTc ratio, and none included a post-treatment ECG time point [5]. This multi-marker gap, combined with the absence of a pain-free period comparison and a matched control group in most available studies, constitutes the primary rationale for the present work.

The aim of this study was to evaluate both atrial and ventricular myocardial repolarization markers in migraine patients presenting to the ED, comparing measurements obtained during the acute attack, after analgesic treatment, and in the pain-free period, and to contrast all findings with those of age- and sex-matched healthy controls.

## 2. Materials and Methods

### 2.1. Study Design and Setting

This prospective observational cross-sectional study was conducted in the ED of Atatürk Sanatoryum Training and Research Hospital, a 780-bed tertiary care facility in Ankara, Turkey, recording approximately 385,000 ED visits annually. The study was approved by the Ethics Committee of Atatürk Sanatoryum Training and Research Hospital (2018, 2012-KAEK-15/1777) and conducted in accordance with the 1964 Declaration of Helsinki and its subsequent amendments. Written informed consent was obtained from all participants before enrolment. Reporting adheres to the STROBE statement for observational studies.

### 2.2. Patient Selection

Between 15 November 2018 and 15 November 2019, patients aged 18–50 years presenting to the ED with an acute headache fulfilling the descriptive criteria for migraine without aura specified by the International Classification of Headache Disorders, 3rd edition (ICHD-3; 1.1, criteria C and D) [18] were screened for inclusion. Criterion C requires at least two of the following pain characteristics: unilateral location, pulsating quality, moderate or severe intensity, or aggravation by routine physical activity. Criterion D requires at least one of the following: nausea and/or vomiting, or both photophobia and phonophobia. Formal aura subtype classification (ICHD-3; 1.1 vs. 1.2) was not recorded at enrolment. The following were excluded: pregnant women; patients with any known cardiac disease (history of coronary artery disease, coronary bypass surgery, or severe mitral or aortic valve stenosis or regurgitation); those using cardiac medications (beta-blockers, nitrates, calcium antagonists, or digoxin); patients with electrolyte imbalances; those with bundle branch block, pathological Q-waves, or left ventricular hypertrophy criteria on ECG; and patients using migraine prophylactic agents with cardiac effects (selective serotonin reuptake inhibitors, propranolol, tricyclic antidepressants, valproate, or calcium channel blockers). Each exclusion criterion was applied to prevent pharmacological or structural confounding of ECG repolarization parameters. Patients who refused to participate were also excluded.

Within 60 min of ED admission, a physical examination was performed and pain intensity was assessed using the Numeric Rating Scale (NRS; 0 = no pain, 10 = maximum pain), with patients classified as having mild (1–3), moderate (4–7), or severe (8–10) pain. Analgesic treatment was administered intramuscularly or intravenously (diclofenac sodium, dexketoprofen, metamizole sodium, or tramadol with normal saline infusion). The second ECG was recorded 60 min after analgesic administration, a fixed interval applied to all patients, contingent on the patient reporting verbal pain relief or a numeric rating scale decrease greater than 4 points. The pain-free ECG was obtained at least 24 h after the attack and within 7 days of the ED visit to minimize residual analgesic effects. An age- and sex-matched control group was recruited from volunteers with no systemic disease and no regular medication use.

### 2.3. Electrocardiography Analysis

Twelve-lead ECGs were obtained using a Cardiofax GEM^®^ (Nihon Kohden, Tokyo, Japan) recorder at a standard acquisition speed of 25 mm/s and amplitude of 10 mm/mV. All ECG images were enlarged to 600 dpi using Adobe Photoshop^®^ (CS3 Lite Version, Adobe Systems Inc., San Jose, CA, USA), an approach validated in the first published Tp-e study on migraine [15], and measured manually. The evaluated parameters were: P-wave duration, P-wave dispersion, QT interval, QT dispersion, QTc duration, QTc dispersion, Tp-e interval, Tp-e dispersion, and Tp-e/QTc ratio. Dispersions were calculated as the difference between the maximum and minimum values across all 12 leads. QTc was calculated using the Bazett formula (QT/√RR), the most widely used correction formula in published migraine ECG studies and endorsed across the relevant comparative literature [19,20,21]; its known tendency to overestimate QTc at elevated heart rates, by a mean of up to 24 ms at heart rates of 80–90 bpm [21], is acknowledged as a study limitation. The Tp-e interval was defined as the time from the peak to the end of the T-wave; the T-wave end was identified using the tangent method, defined as the intersection of the tangent to the steepest descending slope of the T-wave with the isoelectric TP segment [12,22]. T-waves with an amplitude less than 1.5 mm were excluded from measurement [22,23]. The Tp-e/QTc ratio was calculated by dividing the Tp-e interval by the QTc value. All measurements were performed by two emergency medicine specialists who were blinded to each other and to group allocation. Discordant measurements exceeding 20 ms triggered adjudication by a third reviewer [12].

### 2.4. Sample Size

A 15-unit change in QTc dispersion between groups was defined as clinically significant, based on previously published migraine ECG data [15]. With a type-I error of 5%, type-II error of 20% (power 80%), and two-sided analysis, the calculated minimum sample size was 63 per group. Assuming a standard deviation of 30 ms for QTc dispersion, and adding a 10% buffer for protocol attrition, a minimum of 70 patients per group was planned.

### 2.5. Statistical Analysis

Data analysis was performed using SPSS for Windows version 16.0 (IBM Corporation, Armonk, NY, USA). Continuous variables were tested for normality using the Kolmogorov–Smirnov test and presented as medians (interquartile ranges (IQRs), 25th–75th percentiles). Categorical variables are reported as counts and percentages. Between-group comparisons used the Mann–Whitney U test for independent samples; within-patient comparisons across the three ECG time points used the Wilcoxon signed-rank test. Categorical variables were compared using the chi-square test. All reported *p*-values are two-sided, with statistical significance set at *p* < 0.05.

## 3. Results

Seventy migraine patients and 70 healthy controls were enrolled. In the migraine group, 44 patients (62.9%) were female; in the control group, 39 (55.7%) were female (*p* = 0.39). The median age was 28 years (IQR 24–36) in the migraine group and 28 years (IQR 25–35) in the control group (*p* = 1.000). All vital signs and the first ECG in the migraine group were obtained at the ictal time point, at ED presentation and prior to analgesic administration. Systolic blood pressure (120 (113–130) vs. 122 (112–128) mmHg, *p* = 0.721) and diastolic blood pressure (75 (70–80) vs. 75 (73–78) mmHg, *p* = 0.799) did not differ significantly between groups. Pulse rate was significantly higher in the migraine group than in controls (75 (70–83) vs. 70 (65–78.2) beats per minute, *p* = 0.012). Median NRS in the migraine group was 8 (IQR 8–9), indicating predominantly severe pain. Demographic data and ECG parameters (in the migraine group, ictal time point) are summarized in Table 1.

**Figure 1:**
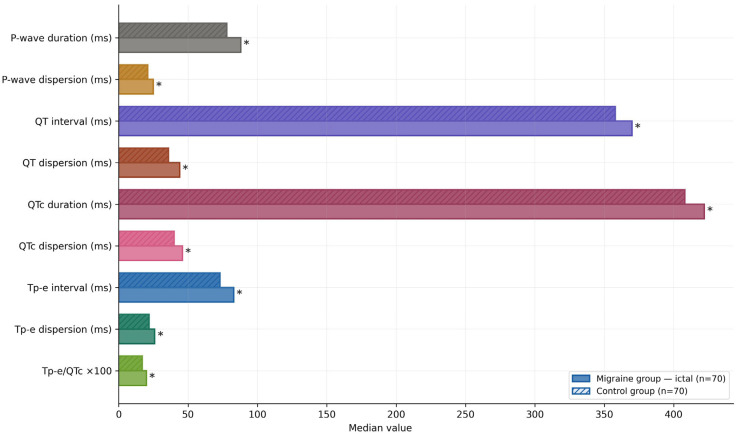
Comparison of ECG repolarization markers between the migraine group (ictal, 1st measurement) and healthy controls. Solid-border bars: migraine group (n = 70); dashed-border bars: control group (n = 70). All between-group differences significant at *p* < 0.05. The statistically superior variable is indicated with an asterisk (*). Values are medians. QTc: Bazett-corrected QT interval; Tp-e: Tpeak–Tend interval.

All repolarization markers were significantly higher in the migraine group than in the control group (*p* < 0.05 for all, Figure 1). P-wave duration (88 (81–93) vs. 78 (72–82) ms, *p* < 0.001), P-wave dispersion (25 (17–29) vs. 21 (16–25) ms, *p* = 0.034), QT interval (370 (357–384) vs. 358 (341.5–362) ms, *p* < 0.001), QT dispersion (44 (32–56) vs. 36 (26–52.5) ms, *p* = 0.034), QTc duration (422.3 (387–446.6) vs. 408 (385.3–431) ms, *p* = 0.002), QTc dispersion (46 (35–63) vs. 40.5 (27–58.3) ms, *p* = 0.003), Tp-e interval (83 (79–87.5) vs. 73 (69–79) ms, *p* < 0.001), Tp-e dispersion (26 (22–31) vs. 22 (18–30) ms, *p* = 0.029), and Tp-e/QTc ratio (0.20 (0.18–0.22) vs. 0.17 (0.15–0.18), *p* < 0.001) were all elevated in the migraine group.

**Figure 2:**
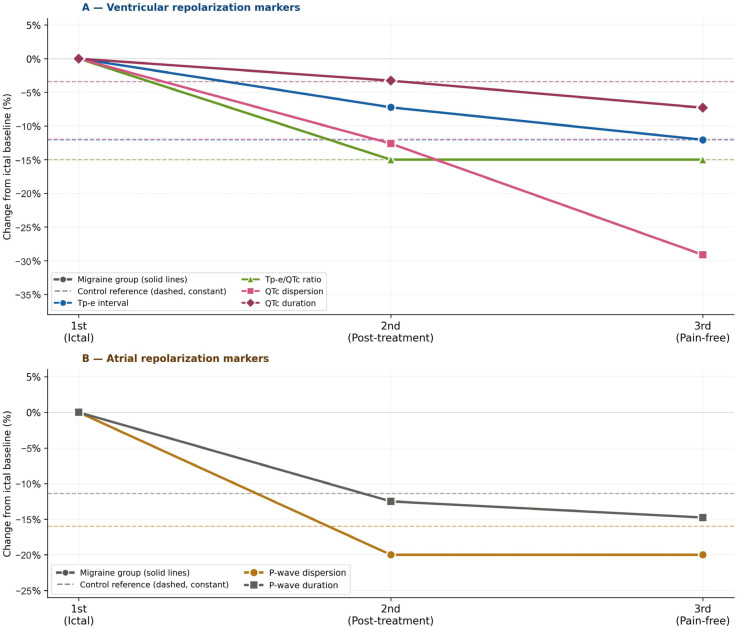
Serial change in ECG repolarization markers from ictal baseline across three time points in the migraine group (n = 70). Solid lines: migraine group data; dashed horizontal lines: control group reference (constant, single measurement). Panel (**A**): ventricular markers. Panel (**B**): atrial markers. QTc: Bazett-corrected QT interval; Tp-e: Tpeak–Tend interval.

Serial measurements within the migraine group across the three ECG time points are presented in Table 2 and Table 3. Comparing the first (ictal) measurement with the second (post-treatment) measurement, all markers except P-wave dispersion decreased significantly (*p* < 0.05 for all). P-wave dispersion was significant for the first-to-second comparison (*p* = 0.034). When the first measurement was compared with the third (pain-free period), all markers were significantly higher during the attack (*p* < 0.05 for all). Tp-e interval and Tp-e/QTc ratio showed a further significant reduction between the second and third measurements (*p* = 0.016 and *p* = 0.004, respectively). P-wave dispersion did not differ significantly between the first and third measurements (*p* = 0.137) or between the second and third measurements (*p* = 0.725). This pattern, in which P-wave dispersion was significant only for the first-to-second comparison and not for the first-to-third or second-to-third comparisons, differed from the trajectory of all ventricular markers. In the migraine group, the serial changes in ECG repolarization markers from the onset of attacks at three time points are detailed in Figure 2.

## 4. Discussion

Acute migraine attacks were associated with significant elevation of both atrial and ventricular myocardial repolarization markers, with near-normalization observed in the pain-free period. The pattern suggests a dynamic, attack-state mechanism rather than fixed structural cardiac change. Tp-e interval and Tp-e/QTc ratio showed a continuous stepwise reduction across all three measurement points, including between the post-treatment and pain-free assessments. P-wave dispersion followed a different trajectory, responding to pain relief but not recovering further in the pain-free period. To our knowledge, prospective emergency department data combining this full complement of atrial and ventricular repolarization markers across three serial ECG time points in migraine patients are limited. These findings carry implications both for emergency physicians monitoring acute migraine patients and for anesthesiologists and reanimation specialists planning perioperative management.

ANS dysfunction is a well-established feature of migraine, characterized by simultaneous impairment of sympathetic and parasympathetic limbs rather than a simple directional deficit [20,24]. Standardized autonomic function testing demonstrates significant impairment across all four major test modalities in migraine patients [4]. The largest effect is seen for sympathetic function during isometric challenge; a consistent, smaller deficit in cardiovagal function during deep breathing is also present. Ictal cardiac autonomic suppression is more pronounced than the interictal deficits captured in cross-sectional testing. Zhang et al. documented a 51% reduction in SDNN during acute migraine attacks compared to the interictal period in 18 episodic migraineurs (56.94 vs. 115.94 ms, *p* < 0.001) [25]. Pain intensity showed a negative correlation with SDNN in that cohort (r = −0.48, *p* = 0.04). Mosek et al., in a prospective autonomic test battery in 17 migraineurs, described resting cardiac sympathetic hypofunction shifting dynamically toward a hyperadrenergic state via cardiovagal withdrawal under orthostatic stress [26]. In our cohort, all repolarization markers were significantly higher during the migraine attack and declined progressively toward control values across the three measurement points. This trajectory is consistent with attack-specific autonomic activation followed by graded recovery. The consistency between our ECG-derived findings and the heart rate variability (HRV) data of Zhang et al. supports the interpretation that repolarization changes reflect genuine sympathovagal fluctuation. Direct simultaneous measurement of HRV indices and ECG repolarization markers across attack phases in a prospective study would confirm this mechanistic link and quantify its magnitude.

QT prolongation and increased QT dispersion during migraine attacks have been documented across multiple independent cohorts, supporting their role as markers of ictal cardiac electrical instability. The only published meta-analysis of ECG parameters in migraine pooled 667 migraineurs and 208 controls across 13 studies [5]. It reported a 6.23-fold higher risk of QTc prolongation during attacks versus pain-free periods (*p* < 0.00001) and a pooled QTc difference of 7.89 standardized mean difference units. Aygun et al. reported a mean ictal QTc of 442 ms in an ED cohort of 30 migraine patients, with 40% exceeding the 440 ms threshold during attacks and none in the pain-free period (*p* < 0.001) [6]. In our study, the median ictal QTc was 422.3 ms (IQR 387–446.6), somewhat lower than the Aygun et al. value, likely reflecting differences in sample composition. Both studies confirm significant ictal QTc elevation with post-attack normalization. QT dispersion in our cohort decreased from 44 ms ictally to 40 ms after treatment. QT dispersion, which reflects regional myocardial repolarization heterogeneity, predicts severe ventricular arrhythmia independently of absolute QTc duration [19]. High inter-study heterogeneity for QTc (I2 = 99%) in the Lee et al. meta-analysis [5] is consistent with methodological variation across included studies. Our standardized 600 dpi acquisition protocol and three-time-point design, absent from all 13 studies in that meta-analysis, reduce this variability and add the recovery dimension that single-time-point designs cannot provide.

The Tp-e interval is established as a marker of global ventricular repolarization dispersion, incorporating both transmural and apicobasal gradients, and its prolongation predicts ventricular tachyarrhythmia and mortality independently of QTc [10,11]. Morin et al. demonstrated in a prospective cohort of 327 patients with left ventricular systolic dysfunction that each 10 ms increment in rate-corrected Tp-e independently predicted ventricular tachyarrhythmia (HR 1.21, *p* < 0.01) and all-cause mortality (HR 1.19, *p* < 0.01) [11]. Patients in the highest Tp-e tertile, above 116.2 ms, faced more than a fourfold increase in arrhythmic risk. The ictal Tp-e values observed in our cohort (median 83 ms, IQR 79–87.5) remained below the 89 ms threshold associated with increased sudden cardiac death risk in the general population [27] and well below the high-risk tertile (>116.2 ms) identified by Morin et al. in left ventricular systolic dysfunction [11]. The clinical significance of the ictal-control difference in our young, structurally normal cohort therefore lies in the demonstrable group-level shift rather than in individual values reaching established proarrhythmic thresholds. Oztürk et al., in the first study measuring Tp-e during migraine attacks, reported an ictal Tp-e of 86.5 ± 7.7 ms in lead V2 versus 75.8 ± 8.1 ms attack-free (*p* < 0.001) in 63 patients [15]. That study used the tail measurement method and identical ECG equipment to ours. Selvi et al. reported an ictal Tp-e of 74.22 ± 20.20 ms versus 65.39 ± 11.33 ms in controls (*p* = 0.001) in an ED-based cohort using the tangent method [17]. QTc did not differ significantly between groups in that study (*p* = 0.612), confirming Tp-e as a more sensitive ictal marker than QTc in the ED setting. Our values are consistent with the Oztürk et al. tail-method measurements. The lower Selvi et al. values are attributable to the known systematic difference between tail and tangent measurement methods. The Tp-e/QTc ratio in our cohort showed a stepwise and statistically significant reduction across all three time points, including between the second and third measurements (*p* = 0.004). This continuing decline beyond analgesic treatment was not reported by either comparator study. The continuing reduction between the post-treatment and pain-free measurements indicates that recovery of ventricular repolarization dispersion follows a gradual temporal course rather than tracking pain resolution alone. Several non-exclusive mechanisms may contribute, including continued decay of analgesic plasma concentrations during the post-treatment window, gradual normalization of sympathetic tone during the postdrome phase, and slower recovery of central autonomic network output relative to the resolution of headache pain. Serial Tp-e monitoring in migraine patients with elevated ictal values and concurrent cardiovascular risk factors would clarify whether this gradual recovery trajectory carries prognostic significance.

P-wave dispersion reflects inhomogeneity of atrial conduction and is an established predictor of paroxysmal AF in conditions associated with increased atrial sympathetic activation [16,24]. Uyarel et al. demonstrated in 726 healthy young adults that state anxiety, operating through sympathetic adrenergic pathways, was the strongest independent determinant of P-wave dispersion, accounting for 66.3% of Pmax variance (*p* < 0.001), with Pmin remaining unchanged [14]. Sarıkaya et al. reported significantly elevated P-wave dispersion in 55 migraine patients compared with 81 healthy controls (133 ± 44 ms vs. 63 ± 23 ms, *p* < 0.001) [16]. Additional prolongation of atrial electromechanical delay was present, confirming a chronic atrial conduction substrate in migraine independent of acute pain. In our cohort, P-wave dispersion was significantly higher in the migraine group than in controls (*p* = 0.034) and declined significantly from the first to the second measurement (*p* = 0.034). It did not differ significantly between the first and third measurements (*p* = 0.137) or between the second and third measurements (*p* = 0.725). This pattern differs from all ventricular markers, which showed orderly stepwise reduction across all three comparisons. The mechanism underlying this atypical trajectory is not fully clear from our data. One plausible explanation is that P-wave dispersion responds primarily to the acute pain-related sympathoadrenergic stimulus, normalizing rapidly after analgesic relief in a pattern analogous to the sympathoadrenergic mechanism described by Uyarel et al. [14], while the chronic atrial substrate described by Sarıkaya et al. [16] prevents further recovery in the pain-free period. The relationship between this atrial conduction substrate and clinically manifest AF requires careful interpretation. The pooled cross-sectional AF prevalence in migraine patients is 1.39%, lower than the 2–4% prevalence reported in older general-population samples [8]; this is consistent with the young age distribution of the included migraine cohorts, in which AF has not yet had time to manifest at population scale. Longitudinal data tell a different story. Among 51,032 migraine patients followed for up to 19 years, Adelborg et al. reported an adjusted hazard ratio for AF or atrial flutter of 1.25 (95% CI 1.16–1.36), with a stronger association in migraine with aura (HR 1.31, 95% CI 1.11–1.55) [9]. The elevated P-wave dispersion observed in our young migraine cohort is consistent with an electrically vulnerable atrial substrate that precedes the longitudinal risk increase captured in older follow-up data. Prospective monitoring with event-based AF detection would clarify whether elevated P-wave dispersion identifies migraine patients at higher risk of progression to clinically manifest AF, particularly those with aura.

Heart rate elevation during migraine attacks reflects sympathetic activation and has direct implications for interpreting rate-corrected repolarization markers. Migraine-associated sympathovagal imbalance drives sinus tachycardia during the ictal period [20]. This sympathetic tone persists between attacks: Kesriklioglu et al. documented sinus tachycardia in 39.5% of migraine patients versus 11.8% of controls during pain-free Holter monitoring (*p* = 0.002) [28]. Pulse rate was significantly higher in the migraine group than in controls in our cohort (*p* = 0.012), confirming ictal sympathetic activation at the group level. This finding is directly relevant to QTc-derived markers. The Bazett formula overcorrects QT at elevated heart rates; Vandenberk et al. demonstrated a mean Bazett–Fridericia difference of 8.8 ms across all heart rates, rising to 24 ms at rates of 80–90 bpm, with Bazett generating 50% more QTc-prolongation alerts than Fridericia for the same dataset [21]. In migraine patients with ictal tachycardia, Bazett-corrected QTc values may overestimate true rate-independent QT prolongation. This limitation applies specifically to QTc duration and QTc dispersion. Tp-e and P-wave dispersion are rate-independent by definition [22]. The Tp-e/QTc ratio addresses this limitation by incorporating rate correction in the denominator, and its continued stepwise reduction across all three time points supports a genuine biological trend rather than a correction formula artefact.

The mechanistic basis for the repolarization changes observed during acute migraine attacks most plausibly involves activation of the ANS through pain-driven engagement of the central autonomic network [29]. Downstream catecholamine-mediated effects on cardiac ion channels then generate the repolarization heterogeneity that our ECG markers reflect. Acute migraine, as an inescapable visceral pain state, routes through the ventrolateral periaqueductal grey to produce sickness-behavior autonomic profile during the attack and sustained sympathetic hyperactivity in the interictal period [3]. The critical cellular intermediary is neural, not humoral. Yagishita et al. demonstrated in a controlled porcine model that direct stellate ganglion stimulation nearly doubled the Tp-e interval and increased whole-heart dispersion of repolarization approximately fourfold [30]. Infusion of norepinephrine at equivalent circulating concentrations produced no change in either parameter. This dissociation establishes that Tp-e elevation reflects heterogeneous regional activation-recovery interval shortening driven by sympathetic nerve activity. Owczuk et al. provided the converse human evidence: selective T1–T4 thoracic epidural blockade reduced TDR from 64.0 to 56.2 ms (*p* = 0.046) and QTcB from 413 to 394 ms (*p* = 0.004) [19]. Lumbar epidural, which spares cardiac sympathetic fibers, produced no such effect. At the ion channel level, adrenergic stimulation modulates the slow delayed rectifier potassium channel (IKs) and the rapid delayed rectifier potassium channel (IKr), the principal repolarizing currents. Heterogeneous sympathetic innervation across endocardial, M-cell, and epicardial myocardial layers prolongs action potential duration unevenly, generating the transmural gradient that the Tp-e interval captures on the surface ECG [12,31]. The same pain–catecholamine–repolarization axis operates outside migraine: Tandogan et al. showed that severe renal colic elevated Tp-e and Tp-e/QTc in an ED cohort, with partial normalization following analgesic treatment [23], mirroring our second-measurement findings. Cortical spreading depression may disturb cardiac autonomic control via insular cortex projections [22], and HRV reduction during attacks [25] indicates that parasympathetic withdrawal amplifies the net sympathetic dominance that widens TDR.

The analgesic agents used in this study carry variable potential for ECG confounding, and this uncertainty requires direct acknowledgement. Diclofenac sodium and dexketoprofen, as non-steroidal anti-inflammatory drugs, carry no significant QTc-prolongation signal at acute analgesic doses. Huang et al. demonstrated in 265,794 UK Biobank participants followed for 12.9 years that acute NSAID use in migraine showed no significant independent cardiovascular disease association [32]. This supports the view that ECG changes in the post-treatment window reflect migraine pathophysiology rather than drug effect. Tramadol lacks clinically relevant QTc prolongation at steady-state therapeutic concentrations [31,33]. To address this directly, we performed a post hoc subgroup analysis comparing the 17 patients (24.3%) who received tramadol with the 53 patients (75.7%) who did not. At the post-treatment time point, no repolarization marker differed between subgroups, including QTc duration (398.3 (387.5–421.0) vs. 409.6 (388.7–420.3) ms, *p* = 0.603), Tp-e interval (75.0 (72.0–85.0) vs. 77.0 (72.0–84.0) ms, *p* = 0.875), and Tp-e/QTc ratio (*p* = 0.745). The two subgroups did not differ at the ictal baseline (lowest *p* = 0.122), which excludes selective tramadol use in patients with more severe ictal repolarization changes. Within the tramadol subgroup, the stepwise reduction in the Tp-e/QTc ratio across the three time points remained significant (1st vs. 2nd *p* = 0.001, 1st vs. 3rd *p* < 0.001, 2nd vs. 3rd *p* = 0.014), tracking the trajectory observed in the whole cohort. These data agree with the pharmacological profile of tramadol and make a tramadol-driven explanation of the observed repolarization changes unlikely. Meperidine carries a less favorable profile. Case reports of QT prolongation and life-threatening ventricular tachycardia with meperidine have been published [31], and it has not been administered to any of our patients. Metamizole sodium has no established QTc signal at analgesic doses, though published data specific to acute intravenous use are limited. Triptans, which carry coronary vasospasm risk through 5-HT1B/1D agonism [24,34], were not administered to any patient, distinguishing our cohort from prior triptan-confounded studies. The third ECG, obtained at least 24 h after the attack and after full analgesic clearance, is the measurement point least susceptible to pharmacological confounding. The significant elevation of all markers at the third measurement relative to controls supports a genuine migraine-related residual effect. Residual uncertainty at the second time point is acknowledged; the post hoc subgroup analysis described above addresses the tramadol confounder specifically, but similar analyses for diclofenac, dexketoprofen, and metamizole were not feasible given their distribution across the cohort.

Cardiac monitoring during acute migraine in the emergency department is not indicated universally but is justified for specific patient subgroups. Pitarokoili et al. documented the only published case of ventricular tachycardia during an acute migraine attack in the ED [7]. The patient was 22 years old with angiographically normal coronary arteries and a structurally intact heart; the self-terminating episode was detected only because of continuous rhythm monitoring. Overt arrhythmia remains uncommon in the predominantly young, structurally intact migraine population. Our data confirm, however, that the arrhythmic substrate, as measured by Tp-e and QTc dispersion, is present during attacks. A 12-lead ECG at ED presentation is indicated in migraine patients with any concurrent cardiovascular risk factor: hypertension, diabetes, a family history of premature sudden cardiac death, or a personal history of syncope. Patients with ictal heart rate above 90 bpm and QTc above 440 ms on admission ECG should receive continuous rhythm monitoring. Tachycardia amplifies both Bazett-related QTc overestimation and the transmural repolarization gradient that Tp-e reflects. Basilar-type migraine warrants particular vigilance. The brainstem autonomic circuits implicated in that subtype overlap with the locus coeruleus and dorsal raphe projections identified in the Pitarokoili case [7]. Patients with migraine, no cardiovascular risk factors, a normal admission ECG, and rapid analgesic response may be reassured: our pain-free period data show near-normalization of all markers. The 385,000 annual ED visits at the study center, combined with the 6.23-fold ictal QTc prolongation risk reported by Lee et al. [5], suggest that systematic ECG recording in at-risk migraine patients would identify a clinically meaningful number with transient arrhythmic vulnerability.

For anesthesiologists and reanimation specialists, the ictal elevation of Tp-e and Tp-e/QTc in structurally normal migraine patients suggests a perioperative repolarization profile that may warrant targeted attention, although the clinical significance of these electrocardiographic differences has not been quantified in outcome data. General anesthesia prolongs QTc in 80% of non-cardiac surgical patients by a mean of 23 ms [35]. Superimposed on the ictal repolarization changes observed in our cohort, this cumulative effect could in principle reach the thresholds associated with proarrhythmic risk, though no study has yet documented this combination in clinical outcome data. On precautionary grounds, scheduling elective surgery during a confirmed pain-free interval may be reasonable in migraine patients with elevated baseline QTc, ictal tachycardia, or established cardiovascular risk factors. This is a precautionary suggestion rather than an evidence-based requirement, and outcome studies will be needed to determine whether timing of elective surgery in relation to migraine attack status influences perioperative arrhythmic events. Preoperative 12-lead ECG with explicit measurement of QTc and, where feasible, Tp-e provides the baseline required for intraoperative risk stratification. If preoperative QTc exceeds 450 ms in men or 460 ms in women, the AHA/ACCF/HRS thresholds [35], anesthetic modification is indicated. Propofol is the preferred induction and maintenance agent, as it does not increase TDR and may attenuate sevoflurane-induced QTc prolongation [34,35]. Desflurane should be avoided: rapid concentration increases trigger a catecholamine and vasopressin surge that prolongs QTc through the identical sympathetic mechanism that elevates Tp-e during migraine attacks [31]. Sevoflurane prolongs QTc via IK inhibition but does not increase TDR uniformly, conferring a lower torsadogenic risk than desflurane or isoflurane [31]. Among antiemetics, ondansetron prolongs QTc and should be replaced by dexamethasone or metoclopramide in patients with elevated preoperative QTc [35]. Thoracic epidural anesthesia covering T1–T4 may have a mechanistic rationale in migraine patients with preoperative Tp-e elevation accompanied by additional proarrhythmic risk factors, given the direct TDR reduction demonstrated by Owczuk et al. with cardiac sympathetic preganglionic blockade [19] and the stellate ganglion stimulation evidence of Yagishita et al. [30]. This remains a speculative extension; no outcome data currently support routine thoracic epidural anesthesia in migraine patients.

Five limitations of this study require explicit acknowledgement. First, the single-center design restricts generalizability to tertiary ED settings comparable to the study site. Tertiary ED patients tend to present with more severe attacks than community migraine patients. The direction of this bias is toward overestimation of repolarization marker elevation. Second, autonomic function was not directly assessed with HRV analysis, baroreflex testing, or tilt-table protocols. The proposed ANS mechanism therefore remains inferential. The true magnitude of sympathovagal imbalance during attacks in this cohort is unknown. Third, the analgesic agents administered carry variable ECG effects. A post hoc subgroup analysis for tramadol (the agent with the most relevant QTc signal in this regimen) showed no significant between-group differences in any repolarization marker at the post-treatment time point. The small tramadol subgroup (n = 17) limits the statistical power of that analysis, and comparable analyses for diclofenac, dexketoprofen, and metamizole were not feasible given their distribution across the cohort. Fourth, several clinical features of the migraine population were not recorded systematically at enrolment: aura subtype classification (with versus without aura), disease duration, attack frequency, and concurrent use of non-excluded prophylactic agents (topiramate and gabapentinoids). Psychiatric history was indirectly addressed through the exclusion of patients receiving selective serotonin reuptake inhibitors (SSRIs) or tricyclic antidepressant treatment, but a formal record of psychiatric diagnoses in remission or untreated comorbid anxiety and depression was not maintained, in line with the limitation already noted regarding the absence of formal anxiety measurement during attacks. The direction of bias from these omissions is uncertain; published evidence suggests that aura has no additional independent cardiac autonomic effect [5], and topiramate and gabapentinoids do not carry clinically relevant QTc signals at therapeutic doses [33]. The exclusion criteria did, however, remove patients with documented cardiovascular disease, electrolyte imbalances, and structural cardiac abnormalities, ensuring that the cohort represented a structurally normal migraine population. Fifth, inter-observer agreement was managed through a 20 ms discordance threshold requiring third-reviewer adjudication, but formal intraclass correlation coefficients were not calculated. Published comparable studies report inter-observer variability of 2.5–3.8% for Tp-e measurement [15]. Whether our protocol achieved equivalent reproducibility cannot be confirmed without ICC data.

## 5. Conclusions

Acute migraine attacks were associated with significant elevation of both atrial and ventricular myocardial repolarization markers in structurally normal patients, with near-normalization in the pain-free period. The Tp-e interval and Tp-e/QTc ratio showed a continuous stepwise reduction across all three ECG time points, including between the post-treatment and pain-free measurements, reflecting a dynamic arrhythmic substrate that persists beyond pain relief but resolves with full sympathovagal recovery. This pattern is consistent with sympathetic nerve activation and catecholamine-mediated TDR during acute migraine, rather than fixed myocardial disease; the mechanism remains inferential and requires confirmation in future studies that directly assess autonomic function alongside repolarization markers. P-wave dispersion declined significantly only between the ictal and post-treatment measurements and showed no further significant change thereafter. This trajectory differed from that of all ventricular markers and is consistent with an atrial substrate that responds rapidly to acute pain-driven sympathoadrenergic stimulation but does not fully normalize in the pain-free interval. The underlying mechanism of this divergent kinetics was not directly tested in the present study. Emergency physicians should consider ECG monitoring and rhythm surveillance in migraine patients with concurrent cardiovascular risk factors or ictal tachycardia. Anesthesiologists managing migraine patients for elective surgery should obtain a pain-free-state ECG preoperatively, prefer propofol-based anesthesia, and avoid desflurane and QT-prolonging antiemetics, and may consider thoracic epidural anesthesia as a precautionary option in patients with preoperative Tp-e elevation accompanied by additional cardiovascular risk factors. Future studies should expand on the present findings in several directions: a prospective multicenter design combining real-time autonomic function assessment, such as heart rate variability and baroreflex sensitivity, with serial ECG repolarization markers and long-term arrhythmia monitoring; subgroup analyses stratified by the specific symptomatic agent administered; and parallel cohorts including patients treated with triptans, so that the pharmacological contribution of each analgesic class to ictal repolarization changes can be quantified separately. Such designs would clarify whether ictal Tp-e and P-wave dispersion elevation translates to hard cardiovascular endpoints in migraine patients.

## Figures and Tables

**Table 1:** Demographic data and ECG repolarization parameters (first measurement) in the migraine patient and healthy control groups.

Parameter	Migraine Group (n = 70)	Control Group (n = 70)	*p*
Sex, female, n (%)	44 (62.9%)	39 (55.7%)	0.39
Age (years)	28 (24–36)	28 (25–35)	1.000
NRS	8 (8–9)	–	-
Systolic BP (mmHg)	120 (113–130)	122 (112–128)	0.721
Diastolic BP (mmHg)	75 (70–80)	75 (73–78)	0.799
Pulse (beats/min)	75 (70–83)	70 (65–78.2)	**0.012**
P-wave duration (ms)	88 (81–93)	78 (72–82)	**<0.001**
P-wave dispersion (ms)	25 (17–29)	21 (16–25)	**0.034**
QT interval (ms)	370 (357–384)	358 (341.5–362)	**<0.001**
QT dispersion (ms)	44 (32–56)	36 (26–52.5)	**0.034**
QTc duration (ms)	422.3 (387–446.6)	408 (385.3–431)	**0.002**
QTc dispersion (ms)	46 (35–63)	40.5 (27–58.3)	**0.003**
Tp-e interval (ms)	83 (79–87.5)	73 (69–79)	**<0.001**
Tp-e dispersion (ms)	26 (22–31)	22 (18–30)	**0.029**
Tp-e/QTc ratio	0.20 (0.18–0.22)	0.17 (0.15–0.18)	**<0.001**

Continuous variables are expressed as medians (IQRs, 25th–75th percentiles), while categorical variables are expressed as frequencies (percentages). Continuous variables were compared using the Mann–Whitney U test, while categorical variables were compared using the Pearson chi-square test or Fisher’s exact test. Statistically significant *p*-values are in bold. In the migraine group, all vital signs and ECG parameters reported in this table correspond to the ictal time point, measured at emergency department presentation prior to analgesic administration. BP: blood pressure; NRS: Numeric Rating Scale; QTc: Bazett-corrected QT interval; Tp-e: Tpeak–Tend interval.

**Table 2:** Serial ECG repolarization measurements in the migraine group at three time points.

Parameter	1st Measurement (Ictal)	2nd Measurement (Post-Treatment)	3rd Measurement (Pain-Free)
P-wave duration (ms)	88 (81–93)	77 (73–81)	75 (71–81)
P-wave dispersion (ms)	25 (17–29)	20 (20–24)	20 (16–28)
QT interval (ms)	370 (357–384)	361 (345.5–374)	358 (347.5–372.5)
QT dispersion (ms)	44 (32–56)	40 (32–44)	40 (26–48)
QTc duration (ms)	422.3 (387–446.6)	408.5 (387.5–421)	391.5 (375.7–421)
QTc dispersion (ms)	46 (35–63)	40.2 (27.7–51.8)	32.6 (26–46.6)
Tp-e interval (ms)	83 (79–87.5)	77 (72–84)	73 (71–83)
Tp-e dispersion (ms)	26 (22–31)	22 (16–28)	21 (16–26)
Tp-e/QTc ratio	0.20 (0.18–0.22)	0.17 (0.16–0.19)	0.17 (0.15–0.18)

Data are presented as medians (IQRs, 25th–75th percentiles). QTc: Bazett-corrected QT interval; Tp-e: Tpeak–Tend interval.

**Table 3:** Pairwise comparisons of ECG repolarization parameters across the three time points in the migraine group (Wilcoxon signed-rank test).

Parameter	*p* (1st vs. 2nd)	*p* (1st vs. 3rd)	*p* (2nd vs. 3rd)
P-wave duration	**<0.001**	**<0.001**	0.087
P-wave dispersion	**0.034**	0.137	0.725
QT interval	**0.004**	**0.002**	0.860
QT dispersion	**0.002**	**0.020**	0.992
QTc duration	**0.032**	**0.012**	0.294
QTc dispersion	**0.004**	**<0.001**	0.164
Tp-e interval	**<0.001**	**<0.001**	**0.016**
Tp-e dispersion	**0.006**	**0.002**	0.460
Tp-e/QTc ratio	**<0.001**	**<0.001**	**0.004**

QTc: Bazett-corrected QT interval; Tp-e: Tpeak–Tend interval. Bold *p*-values indicate significance at *p* < 0.05.

## Data Availability

The datasets generated and/or analyzed during the current study are available from the corresponding author on reasonable request.

## Abbreviations

AF: Atrial fibrillation; ANS: Autonomic nervous system; DOR: Dispersion of repolarization; ECG: Electrocardiogram; ED: Emergency department; HRV: Heart rate variability; IHS: International Headache Society; IQR: Interquartile range; IKr: Rapid delayed rectifier potassium channel; IKs: Slow delayed rectifier potassium channel; LCSD: Left cardiac sympathetic denervation; NRS: Numeric Rating Scale; NSAID: Non-steroidal anti-inflammatory drug; PAG: Periaqueductal grey; Pd: P-wave dispersion; QTc: Corrected QT interval; SDNN: Standard deviation of normal-to-normal RR intervals; TDR: Transmural dispersion of repolarization; Tp-e: Tpeak–Tend interval.
